# Chemical Composition of *Aspidosperma ulei* Markgr. and Antiplasmodial Activity of Selected Indole Alkaloids

**DOI:** 10.3390/molecules18066281

**Published:** 2013-05-29

**Authors:** Zelina Estevam dos Santos Torres, Edilberto Rocha Silveira, Luiz Francisco Rocha e Silva, Emerson Silva Lima, Marne Carvalho de Vasconcellos, Daniel Esdras de Andrade Uchoa, Raimundo Braz Filho, Adrian Martin Pohlit

**Affiliations:** 1 Instituto Nacional de Pesquisas da Amazônia, Caixa Postal 2223 - CEP 69080-971, Manaus, Amazonas, Brasil; E-Mails: zelina@inpa.gov.br (Z.E.S.T.); luizrocha_silva@hotmail.com (L.F.R.S.); 2 Universidade Federal do Ceará, Caixa Postal 12.200 - CEP 60021-940, Fortaleza, Ceará, Brasil; E-Mails: edil@ufc.br (E.R.S.); uchoadea@gmail.com (D.E.A.U.); 3 Universidade Federal do Amazonas, Avenida General Rodrigo Otávio Jordão Ramos, 3000, CEP 69077-000 Campus Universitário, Manaus, Amazonas, Brasil; E-Mails: eslima@ufam.edu.br (E.S.L.); marne@ufam.edu.br (M.C.V.); 4 Universidade Estadual do Norte Fluminense Darcy Ribeiro, CEP 28013-602 Campos dos Goytacazes, Rio de Janeiro, Brasil; E-Mail: braz@uenf.br

**Keywords:** Apocynaceae, indole alkaloids, antiplasmodial, *Plasmodium falciparum* K1, murine fibroblasts, cytotoxic evaluation, NMR, *Aspidosperma ulei*, dasycarpane

## Abstract

A new indole alkaloid, 12-hydroxy-*N*-acetyl-21(*N*)-dehydroplumeran-18-oic acid (**13**), and 11 known indole alkaloids: 3,4,5,6-tetradehydro-β-yohimbine (**3**), 19(*E*)-hunteracine (**4**), β-yohimbine (**5**), yohimbine (**6**), 19,20-dehydro-17-α-yohimbine (**7**), uleine (**10**), 20-*epi*-dasycarpidone (**11**), olivacine (**8**), 20-*epi*-*N*-*nor*-dasycarpidone (**14**), *N*-demethyluleine (**15**) and 20(*E*)-*nor*-subincanadine E (**12**) and a boonein δ-lactone **9**, ursolic acid (**1**) and 1D,1*O*-methyl-*chiro*-inositol (**2**) were isolated from the EtOH extracts of different parts of *Aspidosperma ulei* Markgr. (Apocynaceae). Identification and structural elucidation were based on IR, MS, ^1^H- and ^13^C-NMR spectral data and comparison to literature data. The antiplasmodial and antimalarial activity of **1**, **5**, **6**, **8**, **10** and **15** has been previously evaluated and **1** and **10** have important *in vitro* and *in vivo* antimalarial properties according to patent and/or scientific literature. With the aim of discovering new antiplasmodial indole alkaloids, **3**, **4**, **11**, **12** and **13** were evaluated for *in vitro* inhibition against the multi-drug resistant K1 strain of the human malaria parasite *Plasmodium falciparum*. IC_50_ values of 14.0 (39.9), 4.5 (16.7) and 14.5 (54.3) μg/mL (μM) were determined for **3**, **11** and **12**, respectively. Inhibitory activity of **3**, **4**, **11**, **12** and **13** was evaluated against NIH3T3 murine fibroblasts. None of these compounds exhibited toxicity to fibroblasts (IC_50_ > 50 μg/mL). Of the five compounds screened for *in vitro* antiplasmodial activity, only **11** was active.

## 1. Introduction

Malaria continues to be a disease that afflicts the whole World, especially the African continent. However, data from 99 countries reveals that based on the overall number of deaths malaria is in decline [1]. The main antimalarials available today are the quinolines that are structural mimics of the plant-derived natural product quinine and the semi-synthetic derivatives of another plant-derived natural product, artemisinin. Resistance of the malaria parasites to these drugs is an issue of concern and it is important to discover new compounds that may be developed into the next generation of antimalarial drugs [2].

The *Aspidosperma* spp. (Apocynaceae) comprise trees distributed in Central and South America. *Aspidosperma* spp. extracts exhibit antimalarial activity and remedies prepared from the bark are used in traditional medicine for the treatment of malaria [3]. Screening of bark extracts representing six *Aspidosperma* spp. for *in vitro* inhibition against chloroquine-resistant W2 and chloroquine-sensitive 3D7 strains of the human malaria parasite *Plasmodium falciparum* revealed good activity (IC_50_ = 5.0–65.0 μg/mL). Thus, *A. ulei* (syn. *A. parvifolium*) trunk bark EtOH extracts were found to be active, as were the extracts of two other *Aspidosperma* spp. [4].

Approximately 250 indole alkaloids have been isolated from *Aspidosperma* spp. [5,6,7]. Uleine, 2-methyltetrahydroellipticine, dihydroolivacine and 2-methyltetrahydroolivacine have been previously isolated from *A. ulei* [8,9]. Aspidospermine-type alkaloids inhibit *P. falciparum in vitro* (IC_50_ = 3.2–8.7 μM) [10]. Uleine and uleine-containing extracts have received attention as antiparasitic agents. A plant extract containing uleine as a preventive medication for the treatment of infectious diseases, especially malaria has been patented [11]. Also, standardized extracts of *A. ulei* (cited *A. parvifolium*) containing uleine that exhibit potent antiplasmodial effects against *P. falciparum* [12] have also been patented. There is also experimental evidence that uleine´s pharmacological effects are due to action in the *P. falciparum* digestive vacuole [13]. Other indole alkaloids, aspidocarpine, ellipticine and olivacine isolated from the bark of *A. desmanthum*, *A. vargasii* and *A. olivaceum*, respectively, exhibited significant *in vitro* antiplasmodial activity against the K1 strain of *P. falciparum* [14,15]. Furthermore, ellipticine and olivacine exhibited low cytotoxicity and high *in vivo* antimalarial activity in *Plasmodium berghei*-infected mice at daily doses of 50-100 mg/kg/day in the 4-day suppressive test [15]. 

The aim of the present work was to perform a compositional study on the extracts of *A. ulei* and isolate indole alkaloids from this traditionally used antimalarial plant. Several isolated indole alkaloids were evaluated for *in vitro* antiplasmodial activity and cytotoxicity against fibroblasts as a means to discover new antiplasmodial compounds from this species. 

## 2. Results and Discussion

### 2.1. Isolated Substances from *A. ulei*

**Figure 1:**
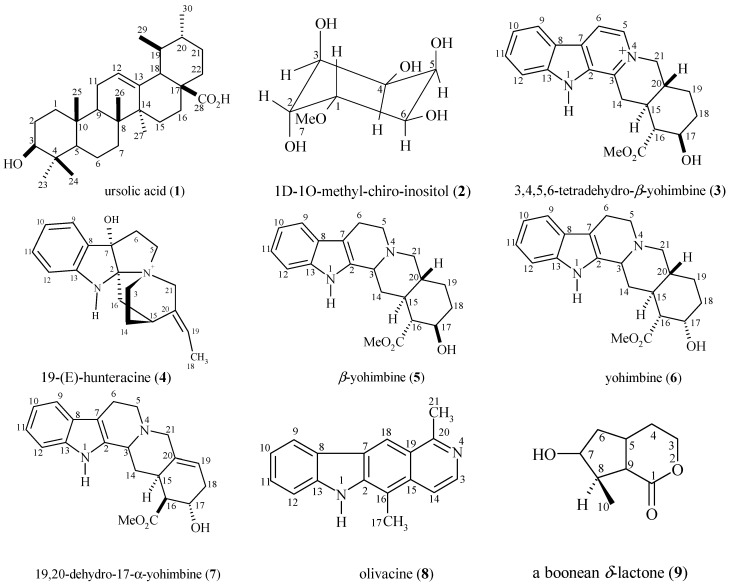

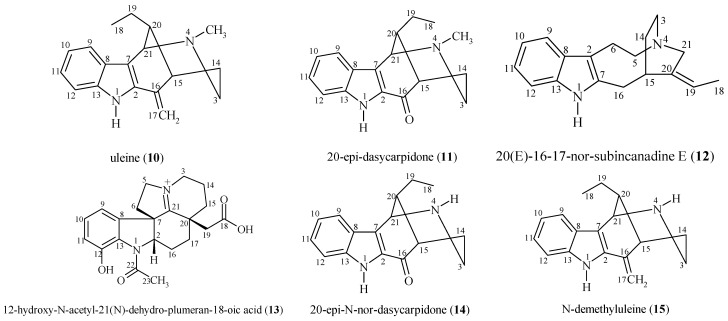
Structures of substances isolated from *Aspidosperma ulei*.

Phytochemical investigation of the leaf, bark, trunk wood, root wood and root bark EtOH extracts of *Aspidosperma ulei* led to the isolation and structural elucidation of several classes of compounds (Figure 1). The indole alkaloids β-yohimbine (**5**) [16], uleine (**10**) [8,9,16], olivacine (**8**) [17], *N*-demethyluleine (**15**) [16] and 20(*E*)-*nor*-subincanadine E (**12**) [16] were isolated in the present work and have been isolated previously from *A. ulei*.

The following known compounds were isolated from *A. ulei* for the first time in the present study: indole alkaloids 3,4,5,6-tetradehydro-*β*-yohimbine (**3**), yohimbine (**6**), 19,20-dehydro-17α-yohimbine (**7**) and 20-*epi*-dasycarpidone (**11**), a triterpene, ursolic acid (**1**), an inositol derivative, methyl-*chiro*-inositol (**2**) and a boonein δ-lactone (6*S*-hydroxy-7*R*-methyl-(4a*S*,7a*S*)-hexahydrocyclopenta[c]pyran-1(3H)-one, **9**). The alkaloids 19*E*-hunteracine (**4)** and 20-*epi*-*N*-*nor*-dasycarpidone (**14**) were isolated and have not been previously reported in a species of *Aspidosperma*. A new indole alkaloid, 12-hydroxy-*N*-acetyl-21(*N*)-dehydro-plumeran-18-oic acid (**13**), was isolated from the root wood of *A. ulei* (Figure 1).

### 2.2. Analysis of Spectral and Physical Data for Isolated Compounds

**Figure 2:**
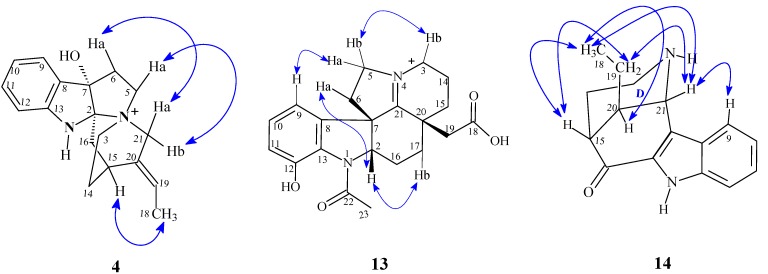
Correlations in the NOESY spectrum for indole alkaloids **4**, **13** and **14** isolated from *A. ulei.*

From the leaf EtOH extract (LEE) of *A. ulei*, ursolic acid (**1**), a white solid, m.p. 296.5–297.6 °C and ${\left[\alpha \right]}_{D}^{20}$ = +26.0° (c. 0.33, MeOH) was isolated for the first time from this species [18,19]. Stem bark EtOH extracts (SBEE) exhibited a precipitate, methyl-*chiro*-inositol (**2**), an amorphous solid, m.p. 150.3–152.2 °C that could be identified based on comparison of its spectral data with that of the literature [20]. Based on acquired spectral data and comparison with data in the literature [21] one of the substances was identified as (+)-3,4,5,6-tetradehydro-*β*-yohimbine (**3**, 25.0 mg), a yellow solid, m.p. 260.0–264.0 °C and ${\left[\alpha \right]}_{D}^{20}$ = +42.3° (c. 0.06, MeOH). In the accurate mass spectrum of this compound there is an H+ adduct signal ([M+H]^+^) at *m/z* 351.1748, that is compatible with the molecular formula C_21_H_22_N_2_O_3_ (theoretical [M+H]^+^ *m/z* 351.1709, Δ = 11 ppm)_._ The indole alkaloid β-yohimbine (**5,** 11.0 and 5.6 mg, respectively) was isolated from SBEE and the root wood EtOH extract basic fraction (RWEEBF) as light yellow-colored needles, m.p. 191.0–192.0 °C, that are soluble in MeOH and CHCl_3_, ${\left[\alpha \right]}_{D}^{25}$ = +12.6° (c. 0.03, MeOH). The MS exhibited a molecular ion adduct ([M+H]^+^) *m/z* 355.28 that is compatible with the molecular formula C_21_H_26_N_2_O_3_. This formula has four Hs more than the quaternary *β*-carboline **3**. The indole alkaloid yohimbine (**6**, 19.0 and 9.8 mg, respectively) was isolated from SBEE and RWEEBF as an amorphous solid, m.p. 226.0–228.0 °C and ${\left[\alpha \right]}_{D}^{25}$ = +57.8° (c 0.90, MeOH). Differences in chemical shifts and coupling constants were observed for H signals assigned to the stereogenic centers and confirmed by ^1^H and ^13^C-NMR and literature data [22], together with data for β-yohimbine (**5**) [22]. Alkaloids **5** and **6** were evaluated for *in vitro* antiplasmodial activity against the chloroquine-resistant Fc M29-Cameroon strain of *Plasmodium falciparum* and found to present IC_50_ values > 1 μg/mL [23]. Several alkaloids, including **6**, were cited in a patent on new antimicrobial agents that included antimalarials [24]. The alkaloid 19,20-dehydro-17α-yohimbine (**7**, 4.0 mg) could be identified by comparison of its NMR data to literature data [25]. It was isolated as an amorphous solid, m.p. 143.2–144.4 °C, ${\left[\alpha \right]}_{D}^{25}$ = +16.8° (c 0.06, MeOH) and exhibited HRMS with signal at *m/z* 353.1892 ([M+H]^+^), that is compatible with the molecular formula C_21_H_24_N_2_O_3_ (theoretical [M+H]^+^ *m/z* 353.1865; Δ = 8 ppm). The aspidospermatane-type indole alkaloid 19*E*-hunteracine (**4**, 5.0 mg) has not been previously isolated from a species of *Aspidosperma*. The IR spectrum of this compound exhibited intense partially overlapped bands centered at 2,924 and 2,853 cm^−1^ that are characteristic of the O-H and N-H stretching band and at 1,673 cm^−1^ a characteristic C=C stretching band. In the ^1^H-NMR spectrum, a signal at *δ*_H_ 5.50 (H-19) was assigned to a vinylic H of a trisubstituted double bond (exocyclic ethylidene group) that was coupled to the H-atoms of a CH_3_ group with signal at *δ*_H_ 1.74 (H-18). H-atoms with signals at *δ*_H_ 3.50 (H-5a and/or H-3b) correlated over two or three bonds with deshielded C-atoms with signals at *δ*_C_ 101.6 (C-2), 88.1 (C-7), 60.1 (C-21) and 43.2 (C-6) are consistent with the presence of a quaternary N-atom bonded to these carbons. Analysis of 2D ^1^H-NMR and ^1^H-NOESY NMR dipolar coupling of H-18 CH_3_ and H-15 (CH) allowed for assignment of the *E*-configuration to the ethylidene group (Figure 2). There are few ^1^H-NMR data available in the literature [26,27,28] for 19*E*-hunteracine which has previously been isolated from *Hunteria eburnea* Pichon (Apocynaceae).

Fractions 4–9 (331.0 mg) and 5–7 (135.0 mg) of the root bark EtOH extract (RBEE) after purification by HPLC furnished a boonein δ-lactone as a brown resin (**9**, 10.0 mg) [29] and the alkaloids uleine (**10**, 40.0 mg) and N-demethyluleine (**15**, 28.0 mg), m.p. 123.0–125.0 °C and 139.9–140.6 °C, respectively. The latter two compounds have been isolated previously from *Aspidosperma ulei*. The structural difference between demethyluleine and uleine is the absence of the N-CH_3_ in the former compound that, according to the literature [30,31,32] leads to differences of *ca*. 2 ppm in chemical shifts depending on the solvents used.

The alkaloids 20-*epi*-dasycarpidone (**11**, 26.0 mg) and 20-*epi*-*N*-*nor*-dasycarpidone (**14**, 13.0 mg), m.p. 164.3–165.3 and 220.3–221.4 °C, ${\left[\alpha \right]}_{D}^{25}$ = −96.0° (c. 0.02, CHCl_3_) and ${\left[\alpha \right]}_{D}^{25}$ +42.7° (c. 0.05, MeOH), respectively, exhibit some differences regarding the chemical shift of C-7 (located on the indole ring). According to literature sources [30,33,34], this carbon should be more deshielded (δc 124.4, 123.8), while in compounds **11** and **14** these carbons are more shielded (δc 113.5 and 116.2). Based on the ^1^H-NMR and ^1^H-NOESY spectra of **14** it was possible to assign the equatorial and axial Hs of the D ring of **14** (Figure 2) that exhibit important dipolar couplings between *δ*_H_ 5.22 (H-21) and 7.85 (H-9), *δ*_H_ 1.35 (H-19) and 0.97 (H-18), couplings at *δ*_H_ 2.87 (H-15), 1.35 (H-19) and *δ*_H_ 0.97 (H-18), besides coupling of *δ*_H_ 2.47 (H-20) and *δ*_H_ 0.97 (3H-18). These data corroborate the epimeric structure of the ethyl side chain in axial position in the piperidine ring. The NMR data obtained for uleine (**10**) and 20-*epi*-dasycarpidone (**11**) provided evidence for the difference of the normal series and *epi* series. In the piperidine ring of uleine the ethyl side chain is in the equatorial position and in 20-*epi*-uleine this side chain is in the axial position. In the spectra of **11**, this difference is evidenced by a 1,3-diaxial γ-effect by the ethyl group on the axial H of C-14, resulting in steric compression of C-14, C-20 and to a lesser extent C-18 and C-19. These C-atoms are more shielded than in the normal series. Olivacine (**8**, 5,0 mg) was isolated from the root bark through precipitation of the root bark EtOH extract acidic fraction (RBEEAF) and exhibited ^1^H and ^13^C-NMR data consistent with those found in the literature [35].

20(*E*)-*nor*-subincanadine E (**12**, 36.0 mg) was isolated from the stem bark of *A. ulei* and its spectral data were similar to those found in the literature [36]. It has been reported as an intermediate in syntheses of *Strychnos* alkaloids [37,38].

The new indole alkaloid 12-hydroxy-*N*-acetyl-21(*N*)-dehydroplumeran-18-oic acid (**13**, 4.4 mg) was isolated as a resin from the root wood EtOH extract (RWEE) of *A. ulei*. The IR spectrum exhibited overlapped broad O-H and N-H stretching bands at 3,440 cm^−1^ and characteristic C=O bands of a conjugated acid and an amide, 1,683 and 1,631 cm^−1^, respectively. In the ^1^H and ^13^C spectra, only three aromatic H signals and three aromatic CH signals were observed. Through long-distance couplings evidenced in the HMBC spectrum it was concluded that the OH group was at the C-12 (δ_c_ 149.2) position thus confirming the monosubstitution of the aromatic ring. Analyses of the HMBC spectrum confirmed the presence of a quaternary N-atom and the C-atom of the iminium (C=N^+^) group (signal at δ_c_ 190.0, C-21) and long-range correlations of *δ*_H_ 4.43 (H-5a), *δ*_H_ 4.07 (H-3a), *δ*_H_ 3.93 (H-3b) and *δ*_H_ 2.29 (H-6b) and *δ*_C_ 190.0 (C-21).

The ^1^H,^1^H-NOESY spectrum of **13** evidenced dipolar couplings between protons at *δ*_H_ 4.68 (H-2) and *δ*_H_ 2.70 (H-6a) and permitted the assignment of the relative stereochemistry of H-2/H-6 as *β*. Also, evident from this spectrum were correlations of signals at *δ*_H_ 3.93 (H-3b) and *δ*_H_ 4.25 (H-5b), at *δ*_H_ 4.68 (H-2) and *δ*_H_ 1.81 (H-17b), and at *δ*_H_ 6.78 (H-9) and *δ*_H_ 4.43 (H-5a) in compound **13**, as shown in Figure 2. A structural similarity search allowed for models to be obtained for comparison of data [39] together with ^1^H and ^13^C data from the literature [40].

### 2.3. *In Vitro* Inhibition of *P. falciparum* and Cytotoxicity

Compounds **1**, **5**, **6**, **8**, **10** and **15** have been evaluated for antiplasmodial activity in previous reports [4,11,14,23,24] and were not tested herein. Antiplasmodial tests were performed on indole alkaloids **3**, **4**, **11**, **12** and **13** herein. Indole alkaloids **7** and **14** were not tested for lack of available material. The results of the evaluation of the inhibitory potential of compounds **3**, **4**, **11**, **12** and **13** *in vitro* against *P. falciparum* are presented in Table 1. To our knowledge this is the first time that the antiplasmodial activity of these compounds was studied. Compounds **3**, **4**, **12** and **13** were inactive (IC_50_ ≥ 11 µg/mL. Compound **11** exhibited antiplasmodial activity [IC_50_ = 4.5 ± 0.2 µg/mL (16.7 μM)]. The cytotoxicity of **3**, **4**, **11**, **12** and **13** was evaluated against NIH3T3 murine fibroblasts. None of these compounds inhibited the growth of fibroblasts (IC_50_ > 50 μg/mL).

**Table 1:** Inhibition of the *in vitro* growth of *P. falciparum* K1 strain by isolated indole alkaloids.

**Nº**	**Name**	**IC_50_ ± SD**	**IC_50_ ± SD**	**Result**
		µg/mL	μM	
**3**	3,4,5,6-tetradehydro-β-yohimbine	14.0 ± 2.7	39.9 ± 7.7	I
**4**	19*E*-hunteracine	> 50.0	> 176	I
**11**	**20-*epi*-dasycarpidone**	**4.5 ± 0.2**	**16.7 ± 0.7**	**MA**
**12**	20(*E*)-*nor*-subincanadine E	14.5 ± 2.8	54.3 ± 10.5	I
**13**	12-hydroxy-*N*-acetyl-21(*N*)-dehydroplumeran-18-oic acid	> 50.0	> 135	I
DS	chloroquine diphosphate	0.17 ± 0.1	0.33 ± 0.19	A
DS	quinine sulphate	0.12 ± 0.05	0.30 ± 0.15	A

SD = Standard deviation, DS = drug standard.. IC_50_ ≤ 0.1 µM = highly active (HA); 0.1 < IC_50_ < 5 µM = active (A); IC_50_ 5–20 µM = moderately active (MA); IC_50_ > 20.0 µM = inactive (I).

## 3. Experimental

### 3.1. General Procedures

Melting points were determined on a Digital Microdetermination apparatus (Mettler Toledo) equipped with a FP82HT heating plate and FP90 processing unit. Determinations were performed at a heating velocity of 2 °C/min and were not corrected. IR spectra were acquired on a Perkin-Elmer Spectrum 100 FT-IR spectrometer using a Universal Attenuated Total Reflectance Accessory (UATR) in the range of 400 to 4,000 cm^−1^. HPLC analysis of calibration solutions and those of extracts and fractions of *A. ulei* was performed on a Waters modular chromatograph. This system was controlled by Empower software. The system consisted of a Waters-1525 binary pump and a photo diode array detector (PDA) model 2996. HPLC separations were performed on a Phenomenex RP-18 column (4.6 × 250 mm, 5 μm) and a Phenomenex RP-18 (10 × 250 mm, 10 μm). The samples were eluted with ACN, MeOH and a solution containing ultrapure H_2_O (Milli-Q, Millipore) and trifluoroacetic acid (TFA, 0.1–0.3%). High-resolution mass spectra (ESI-HRMS) were obtained by dissolving samples in suitable solvents and infusing the resulting solutions directly into the electrospray ionizer of a Shimadzu LCMS-IT-TOF (225-07100-34) mass spectrometer. 1D and 2D ^1^H and ^13^C-NMR spectra such as COSY, HSQC, HMBC and NOESY were obtained on a Bruker Avance DRX500 instrument.

### 3.2. Collection, Botanic Identification and Processing of Plant Materials

*Aspidosperma ulei* is commonly known as pitiá or piquiá. It was collected in Garapa in the City of Acarape in Ceará State, Brazil. Voucher specimens (registry numbers 30823, 32630 and 34813) were deposited in the Prisco Bezerra Herbarium of the University of Ceará. Botanic identification was performed by Prof. Edson P. Nunes of the Department of Biology of the Federal University of Ceará, Fortaleza, Ceará. Leaves, stem bark, heartwood, root bark and root wood were separately dried and milled. Powdered plant materials were weighed and then extracted as described below. 

### 3.3. Preparation of Extracts of *A. ulei* and Isolation Procedures

Extraction of dry, powdered plant materials was carried out by maceration in EtOH at r.t. for 72 h. The mass of each plant material was extracted a total of three times (3 × 10 L). The EtOH solutions obtained from each extraction were rotary evaporated under reduced pressure and combined to provide each extract (Table 2).

**Table 2:** Data for *Aspidosperma ulei* EtOH extract preparation by maceration and evaporation.

Dry, powdered plant material			Dry plant extract			
Part	Mass extracted (kg)		Name	Yield (g)	% Yield	Description
Heartwood	3.0		HWEE	52	1.7	Yellow powder
Leaf	1.0		LEE	98	9.8	Green powder
Root bark	3.0		RBEE	274	9.1	Viscous residue
Root wood	3.0		RWEE	122	4.1	Viscous residue
Stem bark	2.0		SBEE	173	8.7	Viscous residue

HWEE: heartwood EtOH extract, LEE: leaf EtOH extract, RBEE: root bark EtOH extract, RWEE: root wood EtOH extract, SBEE: stem bark EtOH extract.

#### 3.3.1. Isolation of Chemical Components from Leaf Extracts

LEE (10 g) was continuously extracted with Hex, followed by DCM, EtOAc and MeOH providing four fractions after evaporation of solvents. The EtOAc fraction (1.5 g), after normal phase CC (🛇 = 2.5 cm, 8 g of silica gel) using a gradient of increasing polarity of MeOH in CHCl_3_ yielded 37 fractions (10 mL each) of which fraction 7 (59.3 mg) was a finely divided white solid, soluble in CHCl_3_ and MeOH. Spectrometric analysis of NMR, MS and other data revealed this compound to be the pentacyclic triterpene ursolic acid (**1**) reported herein for the first time for this species.

#### 3.3.2. Isolation of Chemical Components from Stem Bark Extracts

SBEE (50 g) was completely dissolved in distilled H_2_O (150 mL) using an ultrasound bath (20 min). Then, DCM (150 mL) was added to yield a 2-phase system. MeOH (100 mL) was added to the H_2_O phase and this mixture was refrigerated for 24 h and yielded a white precipitate (3.35 g) after decantation, that was determined through spectrometric analysis to be the H_2_O soly methyl-*chiro*-inositol (**2**), m.p. 150.3–152.2 °C. CC on the evaporated mother liquor using a gradient of MeOH (5, 10, then 100%) in DCM as eluents yielded 17 fractions (50 mL each). Fractions were combined based on TLC and further purified by preparative HPLC using ACN and 0.2% aq. TFA (70:30), resulting in the isolation of the alkaloid 3,4,5,6-tetradehydro-β-yohimbine (**3**, 25.0 mg).

SBEE (1 g) was dissolved in MeOH and adsorbed onto silica gel (0.5 g) by total evaporation of solvent. The resulting dry silica-sample mixture was fractionated by CC (5.0 g of silica gel, 🛇 = 2,0 cm) by sequential elution with Hex, DCM, EtOAc and MeOH (100 mL of each solvent) followed by total evaporation of fractions. The MeOH fraction (776 mg) was chromatographed on Sephadex LH-20. MeOH was used as eluent. Alkaloids were detected by TLC stained with dragendorff reagent. After sequential chromatographies and purification by semi-preparative reverse-phase HPLC (4.6 × 250 mm, 5 μm) using elution with 0.1% aq. TFA and MeOH (55:45), flow 4.72 mL/min, run time 10 min, detection wavelength 254 nm. 4 fractions were collected that contained, respectively, the alkaloids hunteracine (**4**, 5.0 mg), β-yohimbine (**5**, 11.0 mg), yohimbine (**6**, 19.0 mg) and 19,20-dehydro-17α-yohimbine (**7**, 4.0 mg).

#### 3.3.3. Acid-Base Fractionation of EtOH Extracts

Heartwood EtOH extract (HWEE), RWEE and RBEE (20 g of each) were separately dissolved in 2M HCl (200 mL) with stirring (30 min). Each resulting solution was extracted with DCM (3 × 300 mL). The combined organic phases were dried over anhydrous Na_2_SO_4_, evaporated to dryness and gave rise to the acidic alkaloid fractions of the heartwood, root wood and root bark EtOH extracts (HWEEAF (255 mg), RWEEAF (287 mg) and RBEEAF (384 mg), respectively). Conc. NH_4_OH was added dropwise to each acid fraction until each was pH 9 (Merck 0-14 Indicator Paper). Each fraction was then extracted with DCM (3 × 200 mL). The organic layers were combined, dried over anhydrous Na_2_SO_4_, filtered and totally evaporated to yield basic alkaloid fractions of the heartwood, root wood and root bark EtOH extracts (HWEEBF (363 mg), RWEEBF (302 mg) and RBEEBF (792 mg), respectively).

##### *3.3.3.1. Isolation of Chemical Components from Acidic Fractions* 

RBEEAF was subjected to normal-phase CC (10 g silica gel, 🛇 = 2.5 cm) using a gradient of increasing polarity of MeOH in DCM as eluents and resulting in 12 chromatographic fractions. Chromatographic fractions 4–9 (331 mg) were combined. The alkaloid olivacine (**8**, 5.0 mg) was obtained by precipitation from the combined fraction. The combined fraction was further separated by HPLC using a reverse-phase, semi-preparative column (10.0 × 250 mm, 5 μm) that was eluted using 0.1% aq. TFA and MeOH (45:55). The run time was 15 min at a flow rate of 4.5 mL/min. Six fractions were collected using a detector wavelength of 323 nm. This procedure yielded a boonein lactone (**9**, 10.0 mg) and the alkaloids uleine (**10**, 40.0 mg) and 20-*epi*-dasycarpidone (**11**, 26.0 mg).

The fraction HWEEAF was separated by reverse-phase, semi-preparative HPLC (4.6 × 250 mm, 5 μm) using 0.1% aq. TFA and MeOH (70:30) at a flow rate of 3.0 mL/min, a total run time of 20 min and detector running at a wavelength of 300 nm. Four fractions were collected and fraction 4 (43.0 mg) was sufficiently pure for full spectrometric characterization by 1D and 2D ^1^H and ^13^C-NMR techniques and its structure proved to be that of an indole alkaloid, 20(*E*)-*nor*-subincanadine E (**12**), derived from the stemmadenine skeleton.

##### *3.3.3.2. Isolation of Chemical Components from Basic Fractions* 

RWEEBF was separated by CC (10 g of silica gel, 🛇 = 2.5 cm) using a gradient elution of increasing polarity of MeOH and DCM. 12 fractions were obtained. The combined fraction RWEEBF5-8 (58.0 mg) was separated by semi-preparative, reverse-phase HPLC (10.0 × 250 mm, 5 μm) with elution using 0.1% aq. TFA and MeOH (60:40) at a flow rate of 4.0 mL/min, a run time of 15 min and the detector set at 254 nm. Six fractions were collected that contained the indole alkaloids *β*-yohimbine (**5**, 5.6 mg) and yohimbine (**6**, 9.8 mg) and the new compound 12-hydroxy-*N*-acetyl-21(*N*)-dehydro-plumeran-18-oic acid (**13**, 4.4 mg).

RBEEBF was dissolved in MeOH and adsorbed on 0.5 g of silica gel by pulverization with a mortar and pestle and total evaporation of solvent. The silica-sample mixture was fractionated by CC (2.5 g of silica gel, 🛇 = 2.5 cm) using elution with these solvents: DCM (100%) and then 1, 6 and 100% MeOH in DCM. 10 fractions (25 mL each) resulted. Combined fraction RBEEBF5-7 (135.0 mg) was re-chromatographed using semi-preparative, reverse-phase HPLC (4.6 × 250 mm, 5 μm), elution with 0.1% aq. TFA and MeOH (55:45), and detector operating at 254 nm. The alkaloids 20-*epi*-*N*-*nor*-dasycarpidone (**14**, 13.0 mg) and N-demethyluleine (**15**, 28.0 mg) were obtained from this procedure.

### 3.4. Spectrometric Data for Isolated Compounds

*(+)-3,4,5,6-Tetradehydro-β-yohimbine* (**3**). Yellow solid, m.p. 260.0–264.0 °C, ${\left[\alpha \right]}_{D}^{20}$ = + 42.3° (c. 0.06, MeOH); IR (MeOH) υ _max_ 3344, 3060, 1733, 1637, 1321, 1278, 1166 cm^−1^; ^1^H-NMR (CD_3_OD, 500 MHz): δ 8.36 (*bs*, H-6), 8.25 (*d*, 6.5 Hz, H-9), 8.24 (*d*, 6.7 Hz, H-5), 7.71 (*dd*, 6.5 and 7.0 Hz, H-11), 7.66 (*d*, 8.0 Hz, H-12), 7.38 (*dd*, 6.5 and 7.0 Hz, H-10), 4.79 (*d*, 12.0 Hz, H-21b), 4.32 (*t*, 12.0 Hz, H-21a), 3.84 (*s*, 3H, OCH_3_), 3.82 (*m*, H-17), 3.55 (*dd*, 3.8 and 18.0 Hz, H-14b), 3.18 (*dd*, 10.5 and 18.0 Hz, H-14a), 2.35 (*t*, 10.5 Hz, H-16), 2.21 (*m*, H-15), 1.54 (*m*, H-18a), 2.14 (*m*, H-18b), 2.12 (*m*, H-20), 2.03 (*m*, H-19b), 1.40 (*m*, H-19a). ^13^C-NMR (CD_3_OD, 125 MHz): δ 175.5 (C=O, C-22), 145.4 (C, C-13), 140.5 (C, C-3), 135.5 (C, C-2), 134.0 (CH, C-5), 132.9 (CH, C-11), 132.6 (C, C-7), 124.0 (CH, C-9), 123.2 (CH, C-10), 121.4 (C, C-8), 116.9 (CH, C-6), 113.9 (CH, C-12), 72.3 (CH, C-17), 60.9 (CH_2_, C-21), 58.3 (CH, C-16), 52.7 (OMe, C-23), 36.9 (CH, C-15), 36.5 (CH, C-20), 34.4 (CH_2_, C-18), 31.1 (CH_2_, C-14), 28.2 (CH_2_, C-19).

*19(E)-Hunteracine* (**4**). Yellow solid, m.p. 343.0–343.3 °C; −26.6°, (c. 0.06, MeOH); IV (KBr pellet) υ _max_ 3249, 2924, 1268, 1134, 1673, 1470, 800, 720 cm^−1^. ^1^H-NMR (MeOH, 500 MHz): δ_H_ 7.30 (*d*, 7.0 Hz, H-9), 7.20 (*t*, 7.7 Hz, H-11), 6.92 (*t*, 7.7 Hz, H-10), 6.75 (*d*, 7.7 Hz, H-12), 5.50 (*q*, 9.0 Hz, H-19), 4.55 (*dd*, 2.4, 2.7 Hz, H-21α), 3.95 (*d*, 14.5 Hz, H-21β), 3.75 (*t*, 10.5 Hz. H-3α), 3.50 (*m*, H-5α), 3.37 (*m*, H-5β), 3.37 (*m*, H-15), 3.0 (*m*, H-3β), 2.71 (*d*, 14.0 Hz, H-16α), 2.63 (*m*, H-6α), 2.50 (*m*, H-6β), 2.42 (*m*, H-14α), 2.07 (*dd*, 4.8 Hz, H-16β), 1.98 (*m*, H-14β), 1.74 (*d*, 6.7 Hz, H-18). ^13^C-NMR (MeOH, 125 MHz): δc 147.3 (C, C-13), 133.2 (C, C-8), 131.7 (C, C-20), 131.2 (CH, C-11), 123.9 (CH, C-9), 122.4 (CH, C-10), 119.8 (CH, C-19), 112.2 (CH, C-12), 101.5 (C, C-2), 88.3 (C, C-7), 60.1 (CH_2_, C-21), 57.6 (CH_2_, C-5), 53.7 (CH_2_, C-3), 43.2 (CH_2_, C-6), 34.5 (CH_2_, C-16), 28.0 (CH, C-15), 24.5 (CH_2_, C-14), 12.9 (CH_3_, C-18). ESI-HRMS found: *m/z* 283.1800 [M+H]^+^ (C_18_H_23_N_2_O requires *m/z* 283.1810 [M+H]^+^, Δ = 4 ppm).

*(+)-β-Yohimbine* (**5**). Colorless crystals, m.p. 191.0-192.0 °C; ${\left[\alpha \right]}_{D}^{20}$ = +12.6° (c. 0.03, MeOH); IR (MeOH) υ_max_ 3419, 1726, 1325, 1271, 1060, 742 cm^−1^; ^1^H-NMR (CDCl_3_+CD_3_OD, 500 MHz) δ 7.39 (*d*, 7.8 Hz, H-9), 6.95 (*dt*, 8.0 Hz, H-10), 7.05 (*dt*, 8.0 Hz, H-11), 7.28 (*d*, 8.0 Hz, H-12), 2.90 (*m*, H-6a), 2.74 (*ddd*, 3.2 and 1.6 Hz, H-6b), 3.12 (*dd*, 5.1 and 11.5 Hz, H-5a), 2.63 (*dt*, 4.6 and 11.5 Hz, H-5b), 3.35 (*bd*, 11.0 Hz, H-3), 2.18 (*m*, H-14a), 1.35 (*m*, H-14b), 1.55 (*dt*, 3.0 and 11.0 Hz, H-15), 2.15 (*m*, H-16), 3.81 (*s*, 3H, OCH_3_), 3.78 (*m*, H-17), 2.05 (*ddd*, 3.0, 7.0 and 12.0 Hz, H-18a), 1.40 (*m*, H-18b), 1.70 (*ddd*, 3.0, 7.0 and 12.0 Hz. H-19a), 1.20 (*m*, H-19b), 1.50 (*m*, H-20), 2.97 (*dd*, 3.0 and 11.0 Hz, H-21a), 2.20 (*q*, 11.0 Hz, H-21b).^13^C-NMR (CDCl_3_+CD_3_OD, 125 MHz): δ 177.0 (C=O, C-22), 52.4 (OMe, C-23), 118.8 (CH, C-9), 120.0 (CH. C-10), 122.2 (CH, C-11), 112.2 (CH, C-12), 128.4 (C, C-8), 138.3 (C, C-13), 108.0 (C, C-7), 135.1 (C, C-2), 22.4 (CH_2_, C-6), 54.3 (CH_2_, C-5), 61.5 (CH, C-3), 34.5 (CH_2_, C-14), 43.6 (CH, C-15), 58.9 (CH, C-16), 73.1 (CH, C-17), 35.2 (CH_2_, C-18), 29.2 (CH_2_, C-19), 40.7 (CH, C-20), 61.9 (CH_2_, C-21).

*Yohimbine* (**6**). Amorphous solid, m.p. 226.0–228.0 °C; IR (MeOH) υ _max_ 3404, 3228, 1671, 1370, 1296, 1200, 1124, 739, 719 cm^−1^; ^1^H-NMR (CD_3_OD, 500 MHz): δ 7.49 (*d*, 8.0 Hz, H-9), 7.38 (*d*, 8.0 Hz, H-12), 7.17 (*t*, 7.5 Hz, H-11), 7.07 (*t*, 7.5 Hz, H-10), 4.60 (*d*, 11.4 Hz, H-3), 4.33 (*s*, H-17), 3.82 (*s*, 3H, OCH_3_), 3.76 (*m*, H-5a), 3.50 (*m*, H-5b), 3.50 (*m*, H-21a), 3.24 (*m*, H-6a), 3.08 (*d*, 11.9 Hz, H-6b), 3.08 (*d*, 11.9 Hz, H-21b), 2.85 (*d*, 13.5 Hz, H-14a), 2.46 (*d*, 1.7 Hz, H-16), 2.27 (*m*, H-15), 2.00 (*d*, 2.0 Hz, H-18a), 1.74 (*m*, H-20), 1.74, (*m*, H-18b), 1.64 (*m*, H-19a), 1.59 (*m*, H-14b), 1.59 (*m*, H-19b). ^13^C- NMR (CD_3_OD, 125 MHz): δ 174.7 (C=O, C-22), 138.7 (C, C-13), 130.1 (C, C-2), 127.5 (C, C-8), 123.6 (CH, C-11), 120.8 (CH, C-10), 119.2 (CH, C-9), 112.7 (CH, C-12), 107.0 (C, C-7), 68.3 (CH, C-17), 62.7 (CH, C-3), 59.6 (CH_2_, C-21), 53.8 (CH_2_, C-5), 53.0 (CH, C-16), 52.5 (OMe, C-23), 39.3 (CH, C-20), 35.8 (CH, C-15), 33.2 (CH_2_, C-18), 33.1 (CH_2_, C-14), 23.6 (CH_2_, C-19), 20.4 (CH_2_, C-6). 

*19,20-Dehydro-17α-yohimbine* (**7**). Amorphous solid, m.p. 143.2–144.4 °C; IR (UATR) υ _max_ 3228, 2924, 2854, 1726, 1673, 1455, 1199, 749 cm^−1^, ^1^H-NMR (CD_3_OD, 500 MHz): δ_H_ 7.47 (*d*, 7.8 Hz, H-9), 7.35 (*d*, 7.8 Hz, H-12), 7.15 (*t*, 7.4 Hz, H-11), 7.06 (*t*. 7.4 Hz, H-10), 5.88 (*s*, H-19), 4.45 (*s*, H-17), 4.07 (*m*, H-21a), 3.98 (*m*, H-21b), 3.81 (*s*, 3H, OCH_3_), 3.23 (*m*, H-6a), 3.16 (*m*, H-15), 3.07 (*m*, H-6b), 3.07 (*m*, H-14a), 2.29 (*m*, H-18b), 2.52 (*m*, H-16), 2.52 (*m*, H-18a), 1.52 (*m*, H-14b . ^13^C-NMR (CD_3_OD, 125 MHz): δ_C_ 174.7 (C=O, C-22), 138.0 (C, C-13), 127.4 (C, C-8), 126.8 (CH, C-19), 123.8 (CH, C-11), 120.9 (CH, C-10), 119.3 (CH, C-9), 112.7 (CH, C-12), 106.8 (C, C-7), 66.7 (CH, C-17), 60.1 (CH_2_, C-21), 52.8 (OMe, C-23), 51.4 (CH, C-16), 34.9 (CH_2_, C-14), 34.6 (CH_2_, C-18), 32.9 (CH, C-15), 20.3 (CH_2_, C-6). ESI-HRMS found *m/z* 353.1892 [M+H]^+^ (C_21_H_24_N_2_O_3_ requires *m/z* 353.1865 [M+H]^+^, Δ = 8 ppm).

*Olivacine* (**8**). Yellow solid, m.p. 314.8–315.2 °C; IR (UATR) υ_max_ 3082, 2918, 2851, 1599, 1467, 1409, 1339, 1243, 866, 740 cm^−1^. ^1^H-NMR (MeOH, 500 MHz): δ_H_ 8.84 (*s*, H-18), 8.26 (*d*, 7.4 Hz, H-9), 8.15 (*d*, 6.3 Hz, H-3), 7.90 (*d*, 6.3 Hz, H-14), 7.52 (*m*, H-11/H-12), 7.26 (*m*, H-10), 3.07 (*s*, H-21), 2.81 (*s*, H-17). ^13^C-NMR (MeOH, 125 MHz): δc 160.1 (C, C-20), 144.4 (C, C-13), 141.5 (C, C-2), 137.3 (CH, C-3), 134.6 (C, C-15), 129.3 (CH, C-11), 127.7 (C, C-7), 124.4 (C, C-19), 123.4 (C, C-8), 122.4 (CH, C-9), 120.9 (CH, C-10), 117.2 (CH, C-14), 116.6 (CH, C-18), 112.8 (C, C-16), 112.1(CH, C-12), 21.9 (CH_3_, C-21), 12.6 (CH_3_, C-17).

*Uleine* (**10**). Amorphous solid, m.p. 123.0–125.0 °C; ${\left[\alpha \right]}_{D}^{20}$ = + 9.0° (c. 0.33, CDCl_3_); IR (KBr pellet) υ_max_ 3386, 2926, 1669, 1459, 1198, 1130, 745 cm^−1^. ^1^H-NMR (CDCl_3_, 500 MHz): δ_H_ 8.39 (*s*, N-1), 7.56 (*d*, 10.0 Hz, H-9), 7.35 (*d*, 5.0 Hz, H-12), 7.19 (*dd*, 5.0 and 10.0 Hz, H-11), 7.11 (*dd*, 5.0 and 10.0 Hz, H-10), 5.28 (*s*, H-17a), 5.00 (*s*, H-17b), 4.10 (*d*, 2.0 Hz, H-21), 2.70 (*m*, H-15), 2.49 (*m*, H-3a), 2.30 (*s*, H-5), 2.08 (*m*, H-14a, H-3b, H-20), 1.69 (*m*, H-14b), 1.12 (*q*, 7.0 Hz, H-19), 0.86 (*t*, 7.0 Hz, 3H). ^13^C-NMR (CDCl_3_, 125 MHz): δc 138.9 (C, C-16), 136.8 (C, C-13), 135.4 (C, C-2), 129.6 (C, C-8), 122.9 (CH, C-11), 120.1 (CH, C-10), 119.7 (CH, C-9), 110.9 (CH, C-12), 107.9 (C, C-7), 107.0 (CH_2_, C-17), 56.8 (CH, C-21), 46.5 (CH_2_, C-3), 46.3 (CH, C-20), 44.5 (CH_3_, C-5), 39.7 (CH, C-15), 34.9 (CH_2_, C-14), 24.6 (CH_2_, C-19), 12.0 (CH_3_, C-18). ESI-HRMS *m/z* 267.1877 [M+H]^+^ (C_18_H_22_N_2_ requires *m/z* 267.1861 [M+H]^+^, Δ = 6 ppm).

*(−)-20-Epi-dasycarpidone* (**11**). Amorphous solid, m.p. 164.3–165.3 °C; ${\left[\alpha \right]}_{D}^{20}$ = −96.0° (c. 0.02, CDCl_3_); IV (MeOH) υ_max_ 3383, 3234, 1664, 1466, 1330, 1181, 799, 750, 721 cm^−1^; ^1^H-NMR (CDCl_3_, 500 MHz): δ_H_ 9.88 (*s*, N-H), 7.74 (*d*, 8.0 Hz, H-9), 7.60 (*d*, 8.0 Hz, H-12), 7.51 (*t*, 7.0 Hz, H-11), 7.39 (*t*, 7.0 Hz, H-10), 5.09 (*s*, H-21), 3.37 (*d*, 8.7 Hz, H-3β), 2.88 (*m*, H-3α), 2.86 (*m*, H-15), 2.80 (*s*, N-CH_3_), 2.72 (*m*, H-20), 2.55 (*m*, H-14β), 2.06 (*d*, 15.0 Hz, H-14α), 1.36 (*m*, H-19), 0.91 (*t*, 7.3 Hz, H-18). ^13^C-NMR (CDCl_3_, 125 MHz): δc 190.9 (C=O, C-16), 138.2 (C, C-13), 134.0 (C, C-2), 128.4 (CH, C-11), 126.7 (C, C-8), 123.9 (CH, C-10), 120.7 (CH, C-9), 113.8 (CH, C-12), 113.5 (C, C-7), 58.1 (CH, C-21), 54.3 (CH_3_, C-5), 46.5 (CH_2_, C-3), 46.4 (CH, C-20), 44.2 (CH, C-15), 42.1 (N-CH_3_, C-5), 26.6 (CH_2_, C-14), 24.7 (CH_2_, C-19), 11.3 (CH_3_, C-18) ESI-HRMS found *m/z* 269.1669 [M+H]^+^ (C_17_H_21_N_2_O requires *m*/*z* 269.1654 [M+H]^+^, Δ = 6 ppm).

*20(E)-17-nor-subincanadine E* (**12**). Dark solid, m.p. 230.1–231.2 °C; ${\left[\alpha \right]}_{D}^{20}$ = +76.6° (c. 0.03, MeOH); IV (KBr pellet) υ _max_ 3400, 1677, 1461, 1203, 1132, 800, 721 cm ^−1^. ^1^H-NMR (MeOH, 500 MHz): δ_H_ 12.08 (*s*, N-H), 7.63 (*d*, 8.0 Hz, H-12), 7.54 (*d*, 8.0 Hz, H-9), 7.29 (*dd*, 1.0, 8.0 Hz, H-11), 7.25 (*dd*, 1.0, 8.0 Hz. H-10), 5.58 (*d*, 7.0 Hz, H-19), 4.21 (*d*, 15.0 Hz, H-21α), 3.78 (*d*, 15.0 Hz, H-21β), 3.68 (*dt*, 3.0, 14.0 Hz, H-5α), 3.55 (*ddd*, 3.0, 16.0 Hz, H-6α), 3.44 (*t*, 13.0 Hz, H-5β), 3.25 (*m*, H-16α/β), 3.23 (*dd*, 6.0, 9.0 Hz, H-3α), 3.17 (*m*, H-15), 3.13 (*m*, H-6β), 2.33 (*m*, H-3β), 1.91 (*hept*, 6.0 Hz, H-14α), 1.54 (*d*, 6.0 Hz, H-18), 1.52 (*m*, H-14β). ^13^C-NMR (MeOH, 125 MHz): δ_C_ 136.6 (C, C-13), 136.3 (C, C-2), 135.1 (C, C-20), 128.2 (C, C-8), 124.5 (CH, C-19), 121.8 (CH, C-11), 120.0 (CH, C-10), 118.1 (CH, C-9), 111.9 (CH, C-12), 107.8 (C, C-7), 57.5 (CH_2_, C-5), 52.6 (CH_2_, C-21), 44.8 (CH_2_, C-3), 32.7 (CH_2_, C-16), 30.9 (CH, C-15), 23.1 (CH_2_, C-14), 20.7 (CH_2_, C-6), 13.7 (CH_3_, C-18). ESI-HRMS found *m/z* 267.1900 [M+H]^+^ (C_18_H_22_N_2_ requires *m/z* 267.1861 [M+H]^+^, Δ = 15 ppm).

*12-Hydroxy-N-acetyl-21(N)-dehydroplumeran-18-oic acid* (**13**). Resin, ${\left[\alpha \right]}_{D}^{20}$ = −16.4° (c. 0.05, MeOH); IV (pellet, KBr) υ _max_ 3440, 2943, 1683, 1631, 1475, 1201, 802 cm^−1^. ^1^H-NMR (MeOH, 500 MHz): δ_H_ 7.13 (*d*, 7.9 Hz, H-10), 6.94 (*d*, 7.9 Hz, H-11), 6.78 (*d*, 7.9 Hz, H-9), 4.68 (*m*, H-2), 4.43 (*m*, H-5α), 4.25 (*m*, H-5β), 4.07 (*m*, H-3α), 3.93 (*m*, H-3β), 2.70 (*q*, 10.0 Hz, H-6α), 2.57 (*d*, 16.0 Hz, H-19α), 2.42 (*s*, CH_3_CO-N, H-2’), 2.40 (*m*, H-19β), 2.34 (*m*, H-16α), 2.29 (*m*, H-15α), 2.29 (*m*, H-6β), 2.27 (*m*, H-14α), 2.19 (*m*, H-17α), 2.11 (*m*, H-14β), 1.83 (*m*, H-16β), 1.81 (*m*, H-17β), 1.62 (*m*, H-15β). ^13^C-NMR (MeOH, 125 MHz): δ_C_ 190.0 (C, C-21), 173.5 (C, C-18), 171.9 (C, C-22), 149.2 (C, C-12), 137.0 (C, C-8), 129.7 (CH, C-10), 127.5 (C, C-13), 121.1 (CH, C-11), 115.5 (CH, C-9), 73.2 (CH, C-2), 63.4 (C, C-7), 59.4 (CH_2_, C-5), 51.1 (CH_2_, C-3), 41.7 (C, C-20), 40.9 (CH_2_, C-19), 37.6 (CH_2_, C-6), 33.1 (CH_2_, C-17), 31.1 (CH_2_, C-15), 25.6 (CH_2_, C-16), 22.9 (CH_3_, C-23), 19.3 (CH_2_, C-14). ESI-HRMS found *m/z* 369.1832 [M+H]^+^ (C_21_H_25_N_2_O_4_ requires *m/z* 369.1814 [M+H]^+^, Δ = 5 ppm).

*(+)-20-Epi-N-nor-dasycarpidone* (**14**). Amorphous solid, m.p. 220.3–221.4 °C; ${\left[\alpha \right]}_{D}^{20}$ = +42.7° (c. 0.05, MeOH); IV (pellet, UATR) υ_max_ 3,260, 2,922, 2,852, 1,646, 1,464, 747 cm^−1^; ^1^H-NMR (MeOH, 300 MHz): δ_H_ 7.85 (*d*, 8.0 Hz, H-9), 7.55 (*d*, 7.0 Hz, H-12), 7.44 (*t*, 7.0 Hz, H-11), 7.26 (*t*, 8.0 Hz, H-10), 5.22 (*s*, H-21), 3.17 (*m*, H-3β), 2.92 (*m*, H-3β), 2.87 (s, H-15), 2.47 (*t*, H-20), 2.26 (*m*, H-14β), 2.05 (*bd*, 14.5 Hz, H-14α), 1.35 (*m*, H-19), 0.97 (*t*, 7.0 Hz, H-18). ^13^C-NMR (MeOH_3_, 75 MHz): δc 191.7 (C=O, C-16), 140.4 (C, C-13), 135.5 (C, C-2), 128.6 (CH, C-11), 126.4 (C, C-8), 122.9 (CH, C-10), 122.0 (CH, C-9), 116.2 (C, C-7), 114.5 (CH, C-12), 50.3 (CH, C-21), 47.3 (CH, C-20), 46.6 (CH, C-15), 37.4 (CH_2_, C-3), 26.6 (CH_2_, C-14), 25.6 (CH_2_, C-19), 11.6 (CH_3_, C-18). ESI-HRMS found *m/z* 255.1527 [M+H]^+^ (C_16_H_18_N_2_O requires *m/z* 255.1497 [M+H]^+^, Δ = 12 ppm).

*N-demethyluleine* (**15**). Amorphous solid, m.p. 139.9–140.6 °C; ${\left[\alpha \right]}_{D}^{20}$ = +35.8° (c. 0.05, MeOH); IR (UATR) υ_max_ 3200, 2922, 2853, 1632, 1452, 1321, 737 cm^−1^. ^1^H-NMR (MeOH, 500 MHz): δ_H_ 7.53 (*d*, 7.0 Hz, H-9), 7.34 (*d*, 7.0 Hz, H-12), 7.11 (*t*, 7.0 Hz, H-11), 7.00 (*t*, 7.0 Hz, H-10), 5.53 (*s*, H-17b), 5.01 (*s*, H-17a), 4.36 (*d*, 2.0 Hz, H-21), 2.76 (*s*l, H-15), 2.62 (*m*, 2H-3), 1.99 (*m*, H-14b, H-20), 1.68 (*m*, H-14a), 1.14 (*m*, 2H-19), 0.89 (*t*, 7.7 Hz, 3H-18). ^13^C-NMR (MeOH, 125 MHz): δc 140.0 (C, C-13), 139.2 (C, C-2), 137.2 (C, C-16), 128.0 (C, C-8), 123.7 (CH, C-11), 120.4 (CH, C-10), 119.5 (CH, C-9), 112.2 (CH, C-12), 110.2 (C, C-7), 108.7 (CH_2_, C-17), 50.6 (CH, C-21), 46.7 (CH, C-20), 41.8 (CH, C-15), 37.9 (CH_2_, C-3), 35.7 (CH_2_, C-14), 25.7 (CH_2_, C-19), 12.1 (CH_3_, C-18). ESI-HRMS found *m/z* 253.1709 [M+H]^+^ (C_17_H_20_N_2_ requires *m*/*z* 253.1705 [M+H]^+^, Δ = 2 ppm).

### 3.5. Biological activity of isolated compounds from Aspidosperma ulei

#### 3.5.1. *In Vitro* Culture of *Plasmodium Falciparum* and *in Vitro* Antiplasmodial Assay

The multi-drug resistant K1 strains of *P. falciparum* (Thailand, MRA-159, MR4-ATCC) were maintained in continuous culture [41]. The *in vitro* antiplasmodial test was performed as previously described [14]. Briefly the substances were diluted in DMSO to a stock concentration of 5 mg/mL and subsequently diluted in complete culture medium to obtain sample solutions having concentrations in the range 100-0.14 µg/mL. Sample solutions were applied to the wells of 96-well test plates containing red blood cell suspension having initial parasitemia of 1.5%. Each sample concentration was tested in triplicate and each test plate was incubated for 48 h at 37 °C. After incubation, the contents of the wells were evaluated by optical microscopy. The inhibition of the growth of parasites (IGP%) was evaluated as a percentage by comparison with controls: 

*IGP% = 100 × [1 – (parasitemia with sample/parasitemia of untreated controls)]*

#### 3.5.2. Cell Culture and Cytotoxicity Test Using the Alamar Blue^TM^ Assay

The NHI-3T3 cell line of mouse fibroblasts was grown in DMEN medium supplemented with 10% fetal bovine serum, 2 mM glutamine, 100 µg/mL streptomycin and 100 U/mL penicillin, and incubated at 37 °C with a 5% atmosphere of CO_2_. For assays, the cells were plated in 96-well plates (10^4^ cells per well) and the Alamar Blue^TM^ assay was performed using previously described procedures [42,43]. Briefly, after 24 h, the compounds were dissolved in DMSO and added to each well to give final concentrations of 50 µg/mL. Plates were incubated for 48 h. Control groups had final well concentrations of 0.1% DMSO. Two hours before the end of the incubations, 10 µL of Alamar Blue^TM^ was added to each well. The fluorescent signal was monitored with a multiplate reader using 530–560 nm excitation and 590 nm emission wavelengths.

## 4. Conclusions

This work represents a significant contribution to the knowledge of the chemical composition of *A. ulei*. This included the structural elucidation of a new indole alkaloid, identification of two indole alkaloids not previously reported in *Aspidosperma* spp. and identification of seven known compounds for the first time in *A. ulei*. Isolated indole alkaloid 20-*epi*-dasycarpidone (**11**) was shown to exhibit moderate inhibitory activity against the K1 strain of *P. falciparum*. Furthermore, the presence of highly active antimalarial indole alkaloids olivacine and uleine in *A. ulei* extracts was confirmed in the present study as was the absence of *in vitro* cytotoxicity of several isolated compounds. Taken together, these results lend further support to earlier reports regarding the antimalarial potential of botanicals prepared from *A. ulei* and isolated antiplasmodial and antimalarial components.
